# Effector Protein Translocation by the *Coxiella burnetii* Dot/Icm Type IV Secretion System Requires Endocytic Maturation of the Pathogen-Occupied Vacuole

**DOI:** 10.1371/journal.pone.0054566

**Published:** 2013-01-17

**Authors:** Hayley J. Newton, Justin A. McDonough, Craig R. Roy

**Affiliations:** Department of Microbial Pathogenesis, Yale University School of Medicine, New Haven, Connecticut, United States of America; University of Louisville, United States of America

## Abstract

The human pathogen *Coxiella burnetii* encodes a type IV secretion system called Dot/Icm that is essential for intracellular replication. The Dot/Icm system delivers bacterial effector proteins into the host cytosol during infection. The effector proteins delivered by *C. burnetii* are predicted to have important functions during infection, but when these proteins are needed during infection has not been clearly defined. Here, we use a reporter system consisting of fusion proteins that have a β-lactamase enzyme (BlaM) fused to *C. burnetii* effector proteins to study protein translocation by the Dot/Icm system. Translocation of BlaM fused to the effector proteins CBU0077, CBU1823 and CBU1524 was not detected until 8-hours after infection of HeLa cells, which are permissive for *C. burnetii* replication. Translocation of these effector fusion proteins by the Dot/Icm system required acidification of the *Coxiella*-containing vacuole. Silencing of the host genes encoding the membrane transport regulators Rab5 or Rab7 interfered with effector translocation, which indicates that effectors are not translocated until bacteria traffic to a late endocytic compartment in the host cell. Similar requirements for effector translocation were discerned in bone marrow macrophages derived from C57BL/6 mice, which are primary cells that restrict the intracellular replication of *C. burnetii*. In addition to requiring endocytic maturation of the vacuole for Dot/Icm-mediated translocation of effectors, bacterial transcription was required for this process. Thus, translocation of effector proteins by the *C. burnetii* Dot/Icm system occurs after acidification of the CCV and maturation of this specialized organelle to a late endocytic compartment. This indicates that creation of the specialized vacuole in which *C. burnetii* replicates represents a two-stage process mediated initially by host factors that regulate endocytic maturation and then by bacterial effectors delivered into host cells after bacteria establish residency in a lysosome-derived organelle.

## Introduction

Intracellular bacterial pathogens direct distinct and unique interactions with the eukaryotic host to establish a replicative niche and avoid being killed. Specialized secretion systems that translocate a cohort of virulence proteins called effectors into the host cytosol are often essential for establishing a replicative compartment [1]. These effectors can modulate a wide range of eukaryotic processes, including vesicular trafficking, cytoskeletal dynamics and innate signaling pathways [2]. The Type IVb Secretion System (T4BSS) is a specialized secretion apparatus that delivers effectors to the host [3]. The locus encoding the T4BSS consists of at least 25 genes with several of the components having homology to the *tra/trb* genes from the transfer region of the IncI conjugation plasmid ColIb-P9 of *Shigella sonnei* [4].

A virulence-associated T4BSS system called Dot/Icm was first described in the intracellular pathogen *Legionella pneumophila* and was shown to be essential for replication of *L. pneumophila* inside eukaryotic host cells [5], [6]. The Dot/Icm system is used to modulate host membrane transport pathways and direct the remodeling of the *Legionella*-containing vacuole (LCV) into a unique organelle that interacts with secretory vesicles and supports bacterial replication [7], [8]. The manipulation of host membrane transport pathways by *L. pneumophila* occurs rapidly upon internalization, and intimate contact between the bacterial cell and the host plasma membrane is sufficient to direct the delivery of *Legionella* effectors into the host cytosol even when bacterial uptake is prevented [9]. Thus, the *Legionella* Dot/Icm system is assembled before bacteria engage host cells and initiates effector translocation once cell contact is sensed, which is central to the ability of *Legionella* to rapidly avoid endocytic maturation processes that occur within minutes of bacterial uptake [8].

*Coxiella burnetii* is the causative agent of Q fever, a human infection with a broad spectrum of clinical presentations that range from asymptomatic seroconversion to chronic life-threatening infection [10]. *Coxiella burnetii* is evolutionarily related to *L. pneumophila* [11]. Importantly, *C. burnetii* and a related arthropod pathogen *Rickettsiella grylli* both encode a T4BSS related to Dot/Icm [11], [12]. Several of the *C. burnetii dot* and *icm* genes will restore Dot/Icm-dependent translocation of effectors when produced in *L. pneumophila* mutants deficient in the homologous gene, which indicates these two systems are functionally similar [13], [14].

*Coxiella burnetii* replicates within eukaryotic host cells; however, in contrast to *L. pneumophila*, the *Coxiella*-containing vacuole (CCV) is an acidic lysosome-derived organelle. The normally harsh conditions of the lysosome provide an environment that is essential for intracellular replication of *C. burnetii*. Indeed, early observations demonstrated that *C. burnetii* has minimal metabolic activity when placed in defined medium at pH 7.0, and showed that transport and metabolism of glucose and glutamate is stimulated upon acidification of the medium [15]. The physiological basis for the activation of *C. burnetii* metabolism at low pH is not well understood; however, this observation was vital to recent studies that defined axenic culture conditions for this organism [16], [17].

The ability to culture *C. burnetii* axenically in the medium ACCM-2 at a pH of 4.75 has facilitated genetic studies aimed at identifying bacterial determinants of host pathogenesis [17], [18]. Recent studies have shown that transposon insertions in genes essential for Dot/Icm function result in mutant *C. burnetii* that are incapable of intracellular replication [19], [20]. Presumably, the reliance of both *L. pneumophila* and *C. burnetii* on the Dot/Icm system for intracellular replication reflects the important nature of the cohort of effector proteins delivered into host cells. To date, approximately 60 different *C. burnetii* proteins have been identified as putative effectors based on translocation of these substrates into host cells by the Dot/Icm system [19], [21]–[23]. Over 250 different proteins have been identified as effectors in *L. pneumophila* [24]–[27].

Studies that have examined when effector delivery is first detected after infection of host cells by *L. pneumophila* or *C. burnetii* indicate that there might be important differences in when these two systems are employed. Effector translocation has been studied in both bacteria using a system where the reporter enzyme β-lactamase (BlaM) is fused to an effector, and the fusion protein is expressed constitutively in the bacterial cell using a strong endogenous promoter [23], [28]. In this system the constitutively expressed fusion protein is immediately available for translocation by the Dot/Icm system, which bypasses transcriptional programs that might be used for temporal regulation of effector expression. Translocation of the fusion protein into the host cytosol is then detected using a probe that changes fluorescence when cleaved by the translocated fusion protein. Protein translocation is detected within the first hour of infection when BlaM-effector fusions are produced in *Legionella* [28], which is consistent with studies showing the *Legionella* Dot/Icm system can deliver effectors upon contact with host cells [8]. By contrast, Dot/Icm-dependent translocation of a fusion protein consisting of BlaM fused to the effector CBU0077 could not be detected until eight hours after host cells were infected with *C. burnetii* [19]. This result suggested that the Dot/Icm systems of *Legionella* and *Coxiella* employ different strategies to deliver effector proteins during infection, with the *L. pneumophila* system acting very early and the *C. burnetii* system being initially silent during host cell contact. Here, we investigate cellular events that govern the delivery of *C. burnetii* effectors in an effort to define when the *C. burnetii* Dot/Icm system is activated during infection.

## Materials and Methods

### Bacterial strains and growth conditions

A plaque-purified isolate of *Coxiella burnetii* phase II Nine Mile (NM) strain was propagated in eukaryotic cell lines in Dulbecco's Modified Eagle's Media (DMEM, Invitrogen; Carlsbad, CA) supplemented with 5% FBS (Invitrogen) at 37°C in 5% CO_2_ or ACCM-2 at 37°C in 5% CO_2_ and 2.5% O_2_ as described previously [17]. Chloramphenicol and kanamycin were used in *C. burnetii* ACCM-2 cultures at 3 µg/ml and 300 µg/ml respectively. *C. burnetii* NM and the *icmL*::Tn derivative carrying pBlaM, pBlaM-77 or pBlaM-1524 plasmids were previously described [19]. pBlaM-1823, created by insertion of CBU1823 into the *Sal*I site of pJB-CAT-BlaM, was introduced into *C. burnetii* NM and *C. burnetii icmL*::Tn via electroporation. Expression of BlaM by the above strains was confirmed by western blot using anti-BlaM (1∶5000, QED Bioscience Inc, San Diego, CA). To calculate the multiplicity of infection (MOI) axenically grown *C. burnetii* strains were enumerated by qPCR using *dotA* specific primers [29].

### Preparation of host cells

HeLa 229 cells (CCL-2; ATCC, Manassas, VA) were maintained in Dulbecco's Modified Eagle's Media (DMEM) supplemented with 10% heat inactivated fetal bovine serum (FBS) at 37°C in 5% CO_2_. Bone marrow cells were collected from the femurs and tibiae of C57BL/6 mice and cultured for 7 days in RPMI 1640 containing 20% FBS, 25% macrophage colony-stimulating factor (M-CSF), and penicillin-streptomycin (100 units/ml). Macrophages were seeded 1 day prior to infection in RPMI 1640 containing 10% FBS and 10% M-CSF. Supernatants from L-929 fibroblast cells (ATCC) served as the source of M-CSF. Mice, used as a tissue source only, were maintained in accordance with the guidelines and protocols approved by the Yale Institutional Animal Use and Care Committee (Protocol #07847 to CRR).

### BlaM translocation assay

Translocation of BlaM-effector fusion proteins was performed as previously described [19]. Briefly, 2×10^4^ HeLa cells or 5×10^4^ bone marrow macrophages (BMMs) were seeded in black clear bottom 96 well trays (Corning Incorporated, Corning NY). Approximately 24 h later the monolayers were infected with the appropriate densities of *C. burnetii*. At the desired times post-infection, cells were loaded with the fluorescent substrate CCF4/AM, using the LiveBLAzer-FRET B/G Loading Kit (Invitrogen) with 15 mM probenecid, in the dark for 2 h at room temperature. Translocation was quantified through either microscopy or using a Tecan M1000 plate reader. For single cell assays, cells were visualized by fluorescence microscopy using an excitation of 415 nm and emission at 460 nm and 535 nm. Images were acquired using an inverted Nikon Eclipse TE-2000 S microscope and a 20× objective. At least 300 cells were counted in triplicate wells to determine the percent of cells that were BlaM positive. Images were acquired, exported as TIFF files and labeled with Adobe Illustrator. The magnitude of fluorescent signal was also quantified using the Tecan M1000 plate reader. Following background subtraction the ratio of 460 nm to 535 nm (blue:green) was calculated and expressed relative to uninfected cells. Bafilomycin A (BafA), chloroquine, Brefeldin A (BFA) and rifampicin were added at the time of infection at 100 nm, 100 µm, 1 µg/ml and 10 µg/ml respectively.

### RNA silencing

Small interfering RNA (siRNA) duplexes (Dharmacon; Thermo Fisher Scientific, Lafayette, CO) were diluted in 1x siRNA buffer. A pool of 4 siRNA duplexes was used for transfection with a final concentration of 50 nm. siRNA transfections were carried out with Dharmafect-1 (Dharmacon) in serum-free DMEM according to the manufacturer's protocol. siRNA transfection was performed in black clear bottom 96 well trays with 1.2×10^4^ HeLa cells per well. After three days incubation at 37°C, the siRNA-transfected cells were infected with the *C. burnetii* BlaM reporter strains and incubated for 24 hours at 37°C before translocation efficiency was determined.

### Real-time PCR quantification of gene silencing

Triplicate wells of siRNA treated cells were used to determine the efficiency of RNA silencing. At the time of the translocation assay, 4 days post-transfection of siRNA, HeLa cells were lysed in the presence of DNAse using a Cells-to-Ct Kit (Ambion; Invitrogen) as per the manufacturer's instructions. Reverse transcription was carried out using the Cells-to-Ct kit. Quantitative Real-time PCR was performed from cDNA generated with the kit using iQ™ SYBR® Green Supermix (Bio-Rad Life Science; Hercules, CA) and the following Rab5, Rab7 and Rab1 specific primer pairs. Rab5AF ACCGCCATAGATACACTCTCATC and Rab5AR TCTTTCTGGAGGTAAAGAAACCTG, Rab7AF AGGAAGAAAGTGTTGCTGAAGG and Rab7AR TGATGTCTTCCCGACTCCA and Rab1AF GGGAAAACAATCAAGCTTCAAA and Rab1AR CTGGAGGTGATTGTTCGAAAT. Real Time PCR was performed using an iCycler iQ™ Real-Time PCR Detection System (Bio-Rad). Gene expression levels were normalized to GAPDH expression using the following GAPDH-specific primer pair, GAPDHF AGCCACATCGCTCAGACAC and GAPDHR GCCCAATACGACCAAATCC.

## Results

### The *C. burnetii* Dot/Icm system does not immediately deliver effector proteins into host cells during an early stage of infection

To further investigate the requirements for translocation of effectors by *C. burnetii* we analyzed delivery of the proteins CBU0077, CBU1823 and CBU1524 by the Dot/Icm system. When produced ectopically in eukaryotic cells these three proteins localize to different subcellular compartments, which suggests that they are diverse representatives of the larger cohort of *C. burnetii* effectors [19]. The BlaM protein was fused to the amino terminus of these effector proteins and translocation of the BlaM-effector fusion proteins to the host cytosol by *C. burnetii* was monitored using the fluorescent substrate CCF4-AM. Cleavage of CCF4-AM results in a shift in fluorescence emission from 535 nm (Green) to 460 nm (Blue) when cells are excited at 415 nm. Plasmids encoding *blaM* alone and the *blaM-effector* fusions transcribed by the *C. burnetii* promoter P1169 [18] were introduced into *C. burnetii* NM phase II and the Dot/Icm-deficient control strain *C. burnetii* NM phase II *icmL*::Tn [19]. Immunoblot analysis confirmed that the BlaM and the BlaM-effector fusion proteins were produced by *C. burnetii* grown in ACCM-2, which demonstrates that these proteins are available for translocation before bacteria make contact with the host cell (Figure S1).

**Figure 1:**
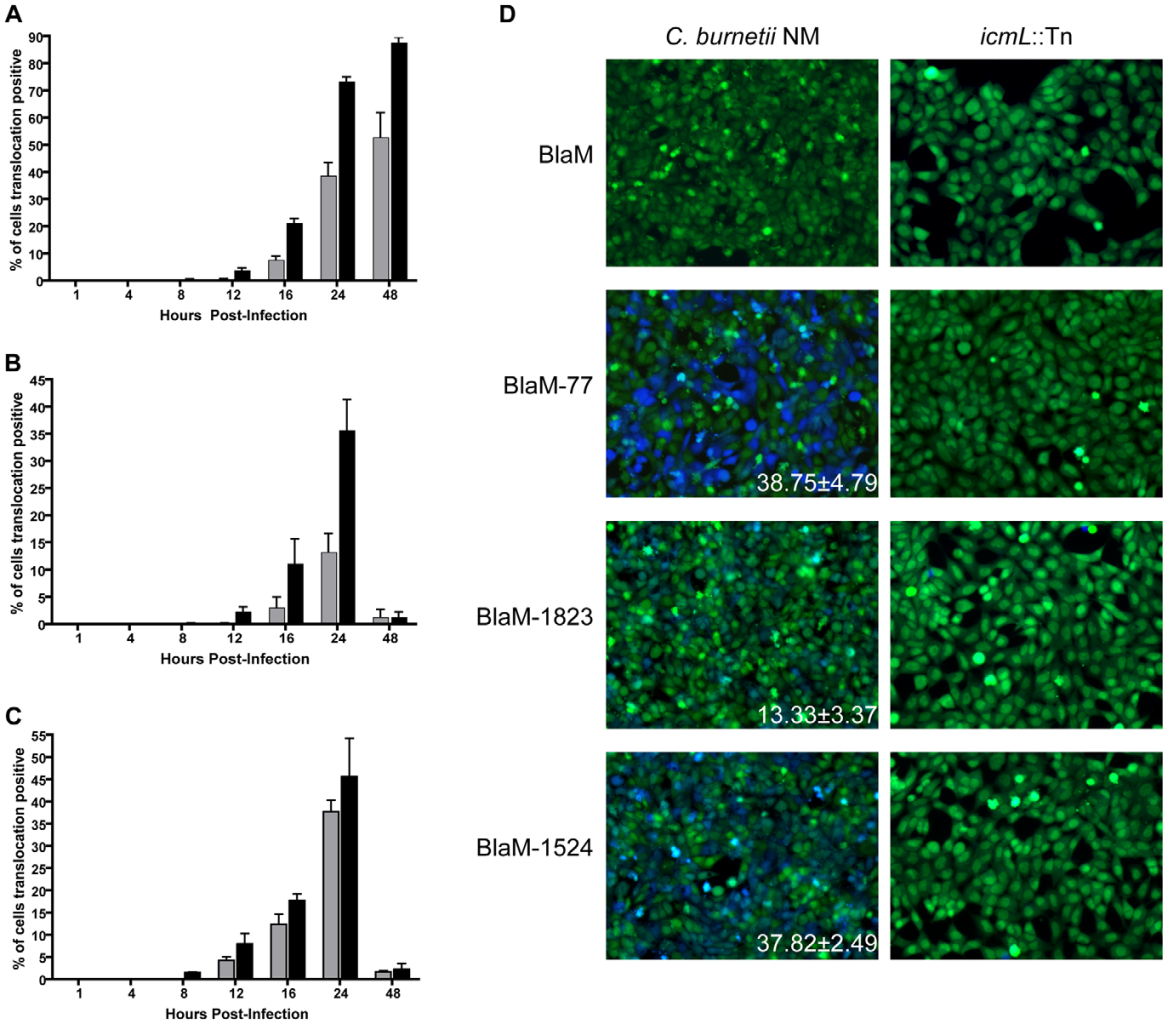
Time course of Dot/Icm dependent translocation. *C. burnetii* NM and the *icmL*::Tn derivative expressing BlaM-77 (A), BlaM-1823 (B) or BlaM-1524 (C) from a plasmid were grown to stationary phase in ACCM-2, enumerated by qPCR, and used to infect HeLa cells at an MOI of 100 (grey bars) and 500 (black bars). At defined times post-infection the BlaM substrate CCF4-AM was added. Low magnification images for each sample were collected and used to calculate the percentage of cells that were translocation positive. This was determined by visual observation of a blue fluorescent emission at 460 nm when the cells were excited at 415 nm. At least 300 cells were quantified per well and each infection was performed in triplicate wells. These experiments were performed at least three independent times. Representative fluorescent micrographs of MOI 100 infections at 24 hours post-infection demonstrate the robust translocation of BlaM-77, BlaM-1823 and BlaM-1524, but not BlaM alone, when expressed by *C. burnetii* NM (D). Green HeLa cells (emission at 535 nm) represent cells loaded with uncleaved CCF4-AM and blue cells (emission at 460 nm) are indicative of CCF4-AM cleaved by translocated BlaM. The mean ± standard deviation percentage of cells that were translocation positive (blue) is displayed in the bottom right corner of each micrograph. No translocation was detected for *C. burnetii* expressing BlaM alone or for *C. burnetii* NM *icmL*::Tn expressing any of the reporter constructs.

HeLa cells were infected with *C. burnetii* producing BlaM alone, BlaM-77, BlaM-1823 or BlaM-1524 at a multiplicity of infection (MOI) of 100 or 500. Fluorescence microscopy was used to identify and enumerate the translocation-positive cells at different times after infection (Figure 1). These data show that the timing of when effector translocation was first detected was similar for the three fusion proteins. Translocation was not detected before eight hours post-infection for any of the fusion proteins, even at the high MOI of 500. This indicates that the delay in translocation previously detected for BlaM-77 is representative of a general delay in the delivery of effectors by the Dot/Icm system of *C. burnetii* and occurs after bacterial uptake by host cells [19]. Although minor variations were detected when translocation efficiencies were compared between the different BlaM-effector proteins, this was not unexpected considering these proteins will have different intrinsic properties that could affect the threshold of detection in this system. Translocation was not detected when the fusion proteins were produced in a Dot/Icm-deficient *icmL*::Tn mutant, which confirms that all of these effectors are delivered by a Dot/Icm-dependent mechanism (Figure 1).

### Vacuole acidification is required for delivery of effectors by *C. burnetii*

**Figure 2:**
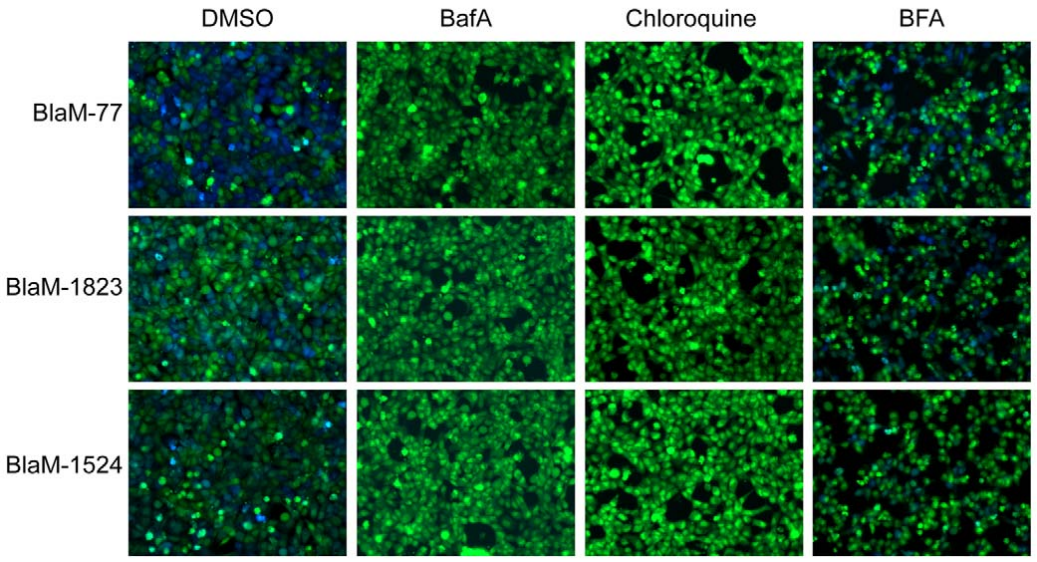
Acidification of the CCV is required for Dot/Icm dependent effector translocation. Translocation assays were performed in the presence of chemical inhibitors to determine host requirements for translocation by the *C. burnetii* Dot/Icm secretion system. HeLa cells were infected with *C. burnetii* NM expressing BlaM, BlaM-77, BlaM-1823 or BlaM-1524 at a MOI of 100 and the infection was allowed to proceed for 24 h before translocation positive cells were quantified. At the time of infection, either BafA (100 nM), chloroquine (100 µM), BFA (1 µg/ml) or an equivalent volume of DMSO were also applied to the cells. DMSO and BFA treatments did not significantly alter the translocation efficiency of the BlaM reporters however no translocation was detected in the presence of BafA or chloroquine. Fluorescent micrographs are representative images of at least three independent experiments.

Because the delay measured in Dot/Icm translocation of the BlaM-effector proteins correlated with the time it would take *C. burnetii* to establish residence in an acidified lysosome-derived organelle, we tested whether vacuole acidification was required for effector translocation using chemical inhibitors that neutralize endosomal pH. Under control conditions, where no inhibitor was added to the cultures, we detected robust translocation of BlaM-77, BlaM-1823 and BlaM-1524 after HeLa cells were infected for 24 hours. By contrast, when acidification of intracellular organelles was blocked using Bafilomycin A (BafA; 100 nM), a specific inhibitor of the vacuolar ATPase, there was no detectable translocation of any fusion protein detected after cells were infected for 24 hours (Figure 2). BafA will neutralize pH gradients that are important for the function of both endosomal and secretory vesicles, so it could not be determined from these data whether the translocation defect detected in BafA-treated cells was due to neutralization of endosomal compartments specifically. Thus, the weak base chloroquine, which neutralizes endosomal compartments following internalization by cells, was used to independently validate the BafA result and to test more specifically whether endosomal neutralization would interfere with effector protein translocation by *C. burnetii*. These data show that the addition of chloroquine (100 µM) to the HeLa cell cultures at the time of *C. burnetii* infection prevented effector protein translocation. The addition of Brefeldin A (BFA; 1 µg/ml), an inhibitor that will primarily affect membrane transport in the secretory pathway, did not significantly alter the efficiency of effector protein translocation (Figure 2). Thus, translocation of effector proteins by *C. burnetii* requires acidification of endosomal compartments.

### Effector protein translocation by *C. burnetii* requires membrane transport to a late endocytic organelle

**Figure 3:**
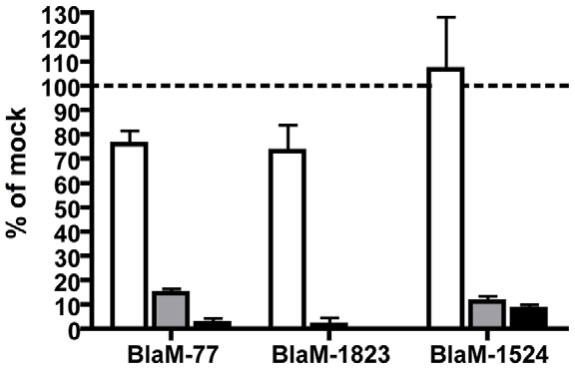
Translocation of Dot/Icm effector proteins is dependent on membrane transport of the vacuole to a late endocytic organelle. HeLa cells were transfected with pools of siRNA specific for Rab1 (white bars), Rab5 (grey bars), Rab7 (black bars) or mock transfected and incubated for three days before being infected with *C. burnetii* BlaM reporter strains at an MOI of 100 for 24 h. Translocation was visually quantified and is presented as the mean ± standard deviation relative to mock-transfected cells from a representative experiment.

Because vacuole acidification is initiated at a very early stage in the endocytic pathway, the neutralization experiments do not clearly define a stage of endosomal maturation that might be necessary to stimulate translocation of effector proteins by the *C. burnetii* Dot/Icm system. To address this question we used siRNA to inhibit distinct stages of endocytic maturation by silencing either Rab5 to interfere with early endosomal maturation events or Rab7 to interfere with late endosomal maturation events [30]. Efficient knockdown of the Rab GTPases was measured by RT-qPCR comparing triplicate samples of mock-treated cells and siRNA-treated cells and using GAPDH transcript levels to normalize results. Gene silencing decreased specific message levels by at least 78% for all siRNA constructs used in these experiments (Figure 3). Importantly, translocation efficiencies for all three effector fusion proteins dropped significantly in cells where Rab5 or Rab7 had been silenced (Figure 3). By contrast, translocation efficiencies were not affected similarly in control cells where Rab1was silenced, which indicates that defects in the transport of early secretory vesicles regulated by Rab1 do not adversely affect translocation of effectors during *C. burnetii* infection. These data indicate maturation of the CCV to a late-endocytic compartment through the activities of Rab5 and Rab7 stimulate delivery of effectors by the Dot/Icm system.

### Translocation of effectors by the *C. burnetii* Dot/Icm system occurs in macrophages that restrict replication

**Figure 4:**
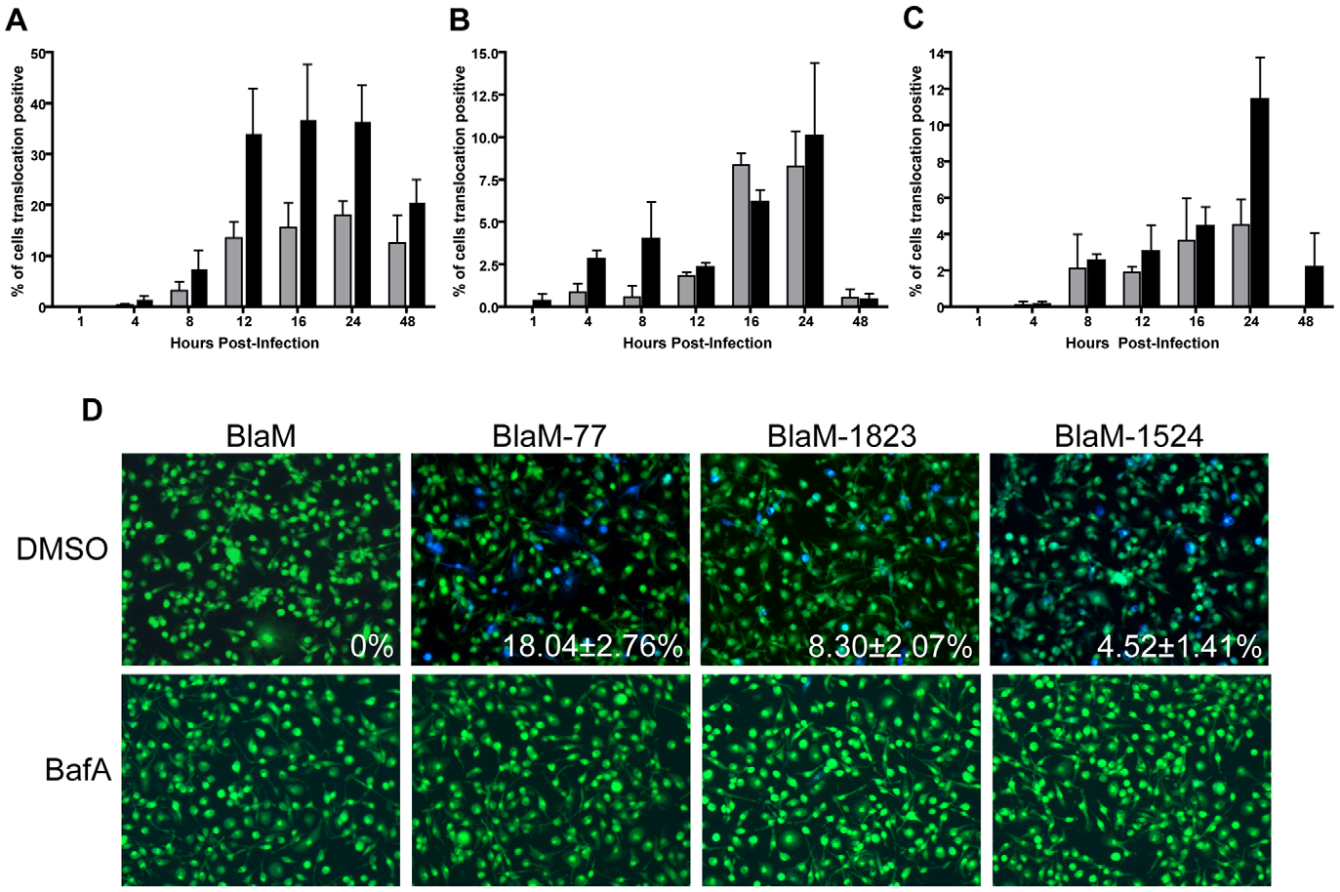
Effector translocation in restrictive bone marrow macrophages. Stationary phase *C. burnetii* NM expressing BlaM-77 (A), BlaM-1823 (B) or BlaM-1524 (C) were used to infect BMMs derived from C57BL/6 mice at an MOI of 100 (grey bars) and 500 (black bars). CCF4-AM was added at the time points shown and low magnification images were analyzed to calculate the percentage of cells that were translocation positive. At least 300 cells were quantified per well and each infection was performed in triplicate wells. These experiments were performed at least three independent times. Representative fluorescent micrographs of MOI 100 infections at 24 hours post-infection demonstrate translocation of BlaM-77, BlaM-1823 and BlaM-1524 (D). The mean ± standard deviation percentage of cells that were translocation positive (blue) is displayed in the bottom right corner of each micrograph. Additionally, the inclusion of 100 nM BafA at the time of infection led to no detectable translocation for any of the reporters (D). No translocation was detected for *C. burnetii* expressing BlaM alone or for *C. burnetii* NM *icmL*::Tn expressing any of the reporter constructs.

Results in HeLa cells that indicate translocation of effectors does not occur before *C. burnetii* has been transported to a lysosome-derived compartment that permits intracellular replication raised the question of whether cells that restrict replication of *C. burnetii* are preventing effector translocation by the Dot/Icm system. This question was addressed using macrophages derived from C57BL/6 mice (BMMs), which restrict the intracellular replication of *C. burnetii* Nine Mile phase II bacteria [31]. BMMs were infected with *C. burnetii* producing BlaM-77 at an MOI of 10 for 24 hours and cells were then scored for intracellular bacteria and for effector translocation. There was clear evidence of effector translocation by *C. burnetii* following infection of BMMs (Figure 4). Single-cell analysis indicated that 97.66±2.52% of the BMMs contained intracellular *C. burnetii* and that 13.09±6.99% were positive for BlaM-77 translocation (Figure S2). The discrepancy between the number of infected BMMs and those that were translocation positive suggests that these non-permissive cells can limit effector translocation by *C. burnetii* but do not block effector translocation entirely. This is in contrast to infection of HeLa cells, where there is a strong correlation between infection and effector protein translocation [19].

We next asked whether the acidification requirements and the temporal delivery of effectors by the Dot/Icm system of *C. burnetii* were similar in non-permissive BMMs and permissive HeLa cells. Similar to the data in HeLa cells, assays in BMMs determined that the three different BlaM-effector fusions were translocated during infection by *C. burnetii* (Figure 4). In BMMs there were a small number of translocation-positive cells detected at four hours post-infection for all three effector proteins, which suggests that the Dot/Icm system is activated sooner following uptake by BMMs. Effector translocation by *C. burnetii* was efficiently blocked when the pH of intracellular organelles was neutralized by treating BMMs with BafA during infection. These data suggest that *C. burnetii* traffics to an acidified compartment more rapidly in BMMs, and that this enables the Dot/Icm system to initiate effector translocation earlier in these non-permissive cells. Thus, the ability of BMMs to restrict intracellular replication of *C. burnetii* is not associated directly with a host mechanism that prevents the Dot/Icm system from becoming functional.

### Bacterial transcription is initially required for translocation of Dot/Icm effector during *C. burnetii* infection

**Figure 5:**
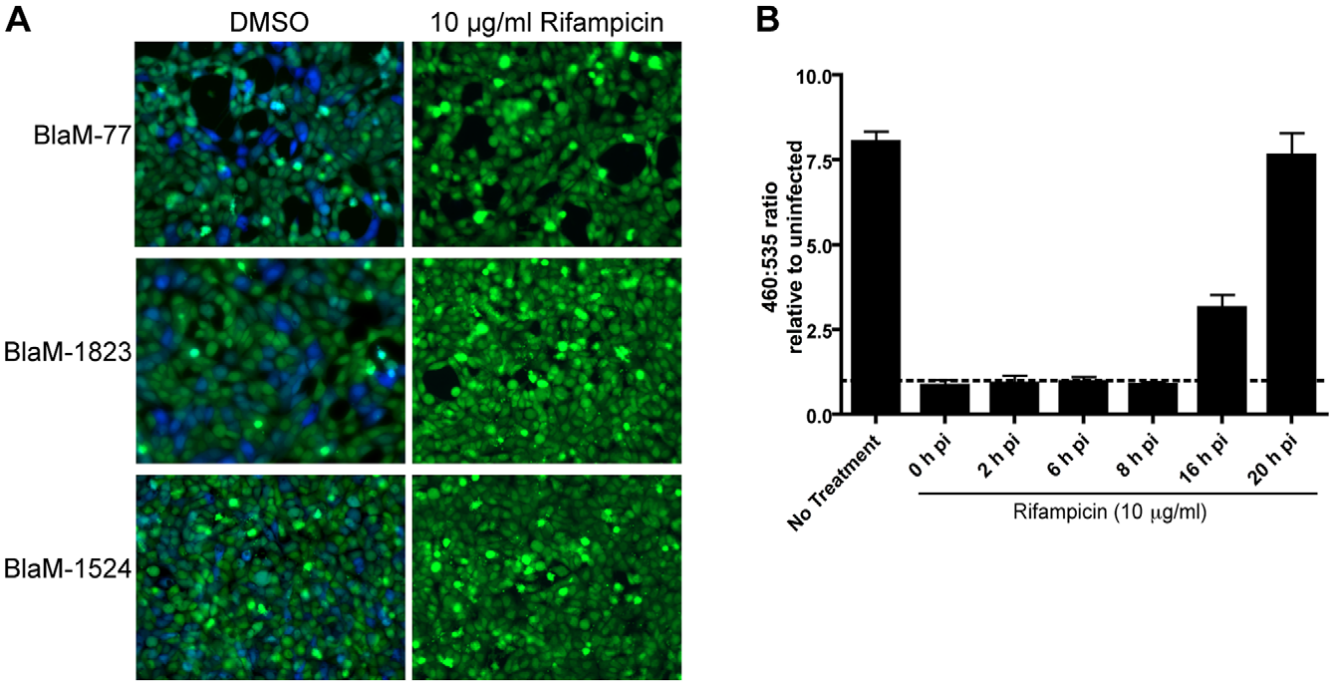
Effector translocation is dependent on *C. burnetii* transcription. Inhibition of bacterial RNA synthesis through the addition of rifampicin blocks the capacity of *C. burnetii* to translocate BlaM-effector reporter proteins. No translocation of BlaM-77, BlaM-1823 or BlaM-1524 is detected after a 24 h infection in the presence of 10 μg/ml rifampicin (A). HeLa cells were infected with *C. burnetii* pBlaM-77 at an MOI of 100 and rifampicin was added at the time of infection, 2, 6, 8, 16 and 20 hours post-infection (h pi) before translocation was measured at 24 h pi (B). Translocation of the BlaM-CBU0077 fusion protein was determined by measuring the change in the 460 nm/535 nm fluorescence emission ratio resulting from cleavage of the CCF4-AM substrate (y-axis). Results represent the mean ± SD obtained from triplicate samples of a representative experiment.

Because the delay in effector translocation correlates the time it would take to deliver *C. burnetii* to an organelle that activates bacterial metabolism, we addressed whether interfering with bacterial transcription would prevent Dot/Icm-mediated translocation of effector proteins. In these studies rifampicin was added at a concentration of 10 μg/ml at the time of infection to prevent bacterial transcription, and infections then proceeded for 24 hours before translocation was measured. Effector translocation was not affected when cells were treated with the solvent DMSO alone. When rifampicin was added at the time of infection, however, effector translocation was blocked (Figure 5A). Rifampicin was added to infections at different times after infection to determine more precisely the stage at which preventing bacterial transcription would prevent effector translocation. These data showed that the addition of rifampicin during the first eight hours of infection effectively blocked effector translocation (Figure 5B). Thus, there is close correlation between the time when *C. burnetii* becomes metabolically active inside host cells and when rifampicin prevents effector translocation.

## Discussion

The molecular strategies used by *C. burnetii* to coordinate the establishment of a vacuole that supports intracellular replication are not clearly understood. The recent development of axenic culture conditions and genetic approaches to study *C. burnetii*, however, enabled new approaches to be used to address fundamental questions related to the mechanism of host infection [17], [32]. Isolation of *C. burnetii* mutants with a transposon insertion inactivating the genes *icmL* [19] or *icmD* [20] demonstrated that the *C. burnetii* Dot/Icm T4BSS is essential for intracellular replication. This mirrors the replication defect seen for *L. pneumophila* mutants deficient in Dot/Icm activity [5], [6]. In *L. pneumophila*, the Dot/Icm system must be functional at the earliest stage of infection [8]. Similar experiments were conducted in *C. burnetii* and it was shown that when a gene essential for Dot/Icm function was put under the control of an inducible promoter, the Dot/Icm system could be activated 24-hours after infection and *C. burnetii* replication was restored [20]. Thus, unlike the *L. pneumophila* Dot/Icm system, these data suggest that the Dot/Icm system in *C. burnetii* is not essential until a late stage of infection.

One of the primary aims of effectors translocated by the *L. pneumophila* Dot/Icm system is to disrupt endocytic trafficking of the phagosome and to promote remodeling of the LCV by host secretory vesicles. Given that *C. burnetii* replicates in a vacuole with a low pH and the degradative capacity of a lysosome [33], it is not obvious how effectors of the *C. burnetii* Dot/Icm system mediate changes upon the lysosome-derived CCV to convert it to a replication-permissive environment for *C. burnetii* [34]. Thus, more research is needed to define the different aspects of CCV development that the Dot/Icm system impacts. Towards this end we set out in this study to address when effectors are translocated by the Dot/Icm system, which would provide further insight into the stage of infection that effector proteins must function to promote intracellular replication of *C. burnetii*.

We had previously reported that Dot/Icm-mediated translocation of BlaM-77 was first detected at roughly eight-hours post-infection in a HeLa cell infection assay [19]. Here, we have extended these preliminary results with data demonstrating that two other *C. burnetii* effectors, selected because they display different intracellular localization phenotype than CBU0077, were translocated with similar temporal parameters as CBU0077. Immunoblot analysis demonstrated that these fusion proteins were all produced by *C. burnetii* cultured in the ACCM-2 medium and that were used to infect the eukaryotic cells. Thus, the delay in detection of translocation for all three effector fusion proteins suggested that unlike the *L. pneumophila* Dot/Icm system, which is designed to inject effectors immediately upon contact with the host cell, the *C. burnetii* system is regulated in a different manner and is designed to inject effectors at a later stage of host infection.

It was not unexpected to find differences in the efficiency of translocation of BlaM-77, BlaM-1823 and BlaM-1524. The threshold by which translocation of an effector will be detected at the single cell level in the BlaM assay will be influenced by a number of intrinsic factors that will vary in all effector fusions. These factors include the strength of the signal sequence in the effector that is recognized by the Dot/Icm system, the half-life of the individual fusion protein in the host cytosol, the specific enzymatic activity of the BlaM-fusion protein, and the localization properties of the effector protein inside the host cytosol. It is important to note, however, that despite all of the intrinsic factors that will influence the threshold of detection of an individual effector using the BlaM system, the timing of translocation was similar for all three effector fusion proteins, with all three effector fusions displaying a delay of roughly eight hours after infection before translocation was detected. Importantly, the temporal pattern detected was not influenced simply by increasing the MOI of the infection from 100 to 500. Changing the MOI to 500 should have increased the amount of effector being delivered into each cell, which theoretically should have reduced the time of detection by roughly 80% compared to an MOI of 100 if the amount of effector in the cytosol was the major determinant limiting detection. Thus, these studies strongly implicate that the *C. burnetii* Dot/Icm system is not fully functional when bacteria come in contact with host cells, and that translocation of pre-synthesized effectors is tightly regulated during the infection processes.

The hypothesis that effector proteins are not secreted during the early stages of *C. burnetii* infection of host cells was validated independently using chemical and genetic inhibitors to disrupt different stages of CCV maturation. Previous studies had demonstrated that a mature, acidified CCV is essential for intracellular replication of *C. burnetii*. Specifically, neutralizing the pH of intracellular organelles with BafA or chloroquine was shown to prevent intracellular replication of *C. burnetii* [35], and the expression of dominant-negative mutants of Rab5 and Rab7 in host cells was shown to interfere with *C. burnetii* intracellular replication [36]. Data presented in this current study show that these same conditions are effective at preventing the translocation of effector proteins by *C. burnetii*, which provides a clear link between host events that are required for intracellular replication and the delivery of proteins into the host cell by the Dot/Icm system.

Interfering with bacterial transcription by adding rifampicin during the early stages of infection was also found to interfere with effector translocation by *C. burnetii*. This result implies that early gene transcription events are important for Dot/Icm-mediated translocation of effectors by *C. burnetii*. This may mean that there are bacterial factors that must be synthesized after *C. burnetii* infect host cells that are essential for activation of the Dot/Icm system. Consistent with this possibility it has been shown that several *dot* and *icm* genes are upregulated during the first eight hours of host cell infection, which suggests that essential components of the Dot/Icm apparatus may not be immediately available upon bacterial uptake [37]. It is also possible that by inhibiting transcription at an early stage, the bacteria remain metabolically dormant. In *L. pneumophila* it has been shown that inhibitors that collapse the proton motive force and disrupt ATP generation in the bacterial cell will also interfere with effector translocation by the Dot/Icm system [38]. Thus, the mechanism by which rifampicin prevents effector translocation during *C. burnetii* infection is likely complex and multifactorial; however, the rifampicin data correlates with the other assays that indicate Dot/Icm-mediated translocation of effectors during host cell infection is tightly linked to delivery of the bacteria to a compartment that promotes bacterial metabolism.

Although it remains possible that *C. burnetii* has a subset of Dot/Icm effectors that can be translocated by dormant bacteria during the initial stages of infection, this seems somewhat unlikely. We base this argument on the fact that the BlaM-effector proteins being measured for translocation are overexpressed in the bacterial cell and would be at a competitive advantage over other effectors for engaging a functional secretion system. Even if these effector fusion proteins have a lower affinity for the apparatus than putative early effectors expressed endogenously, the overexpression of the fusion proteins would be expected to override this biochemical difference in affinities. Additionally, similar delays in effector translocation were measured for the three effectors selected having different intrinsic properties, suggesting the delay is a general feature indicating that the Dot/Icm system is inactive during the early stages of infection. Most importantly, in the *L. pneumophila* system, all of the 270 effectors that have been validated as substrates of the Dot/Icm apparatus appear to be translocated into host cells with similar kinetics when produced ectopically in the bacterial cell. Thus, if they exist, *C. burnetii* effectors that might be translocated during the very early stages of infection would likely have features that are unique for T4BSS effectors.

Given that Dot/Icm secretion is essential for intracellular replication of *C. burnetii*, we addressed whether the ability of non-permissive macrophages to restrict intracellular replication of *C. burnetii* was associated with a strong defect in Dot/Icm-mediated translocation of effectors. These experiments revealed translocation of effectors could not be detected in a proportion of BMMs that were infected with *C. burnetii*; however, translocation was evident in a significant number of infected BMMs. This implies that BMMs can restrict replication of *C. burnetii* that have been successful at delivering effectors into the host cytosol. These data correlate with studies suggesting that *C. burnetii* NM phase II bacteria have a survival defect inside of BMMs and are degraded within the lysosome-derived vacuole, which is probably related to a defect in the bacterial cell envelope. It was intriguing to find that the delay in detecting Dot/Icm-mediated effector translocation was shorter following *C. burnetii* infection of BMMs compared to HeLa cells even though bacterial survival and replication are impaired in these cells. The shorter delay in effector translocation detected in BMMs likely reflects the fact that macrophages are efficient at rapidly transporting bacteria to lysosomes, which would mean *C. burnetii* gains access to their preferred niche more rapidly when internalized by BMMs and this results in the Dot/Icm-system becoming functional sooner. These data provide additional evidence that the BlaM translocation assay is not limited primarily by the number of molecules of effector proteins delivered into the cytosol, as decreased intracellular survival should result in fewer molecules of a BlaM effector being delivered into the cytosol by each bacterium, yet the time it took to detect protein translocation was shorter in the BMMs.

The observation that the Dot/Icm system is not active throughout the early stages of infection provides additional insight into the possible roles of the Dot/Icm system during *C. burnetii* infection. Because effector translocation does not occur until the CCV has matured into an acidified lysosome-derived compartment, the Dot/Icm system is unlikely to play a role in modulating the early events during CCV transport.

Thus, the prediction is that some of the effector proteins of the *C. burnetii* Dot/Icm system will be important for converting the acidified lysosome-like compartment that is derived from host-mediated transport events into a specialized vacuole that enables proliferation of *C. burnetii* intracellularly. Effectors are likely to promote the fusogenic properties of the vacuole that result in a spacious CCV [39] and the strong anti-apoptotic phenotype displayed during infection of mammalian host cells by *C. burnetii* [40], [41].

As suggested previously [34], there may be an advantage to delaying effector translocation until *C. burnetii* has reached a lysosome. In *L. pneumophlia*, the rapid translocation of Dot/Icm effectors into the host cytosol is associated with a robust innate immune response mediated by cytosolic pathogen sensors [42]. It is feasible that *C. burnetii*, a mammalian adapted pathogen, employs tight regulation of Dot/Icm secretion as a mechanism to evade early innate immune detection by the host. Thus, understanding how *C. burnetii* regulates effector translocation by the Dot/Icm system may provide new insight into how this organism is able to modulate host inflammatory responses and persist inside mammalian hosts.

## Supporting Information

Figure S1 **BlaM-effector fusion proteins expressed in** ***C. burnetii*** **.** Immunoblot analysis of *C. burnetii* NM phase II and the *icmL*::Tn mutant following the introduction of pJB-CAT-BlaM (BlaM) and BlaM-effector fusion constructs (pBlaM-77, pBlaM-1524 and pBlaM-1823). Lysate from stationary phase ACCM-2 cultures were probed with anti-BlaM (1∶5000) and the highlighted bands demonstrated expression of BlaM (29 kDa), BlaM-77 (60 kDa), BlaM-1524 (54 kDa) and BlaM-1823 (70 kDa). Importantly, expression of each reporter protein was comparable in *C. burnetii* NM phase II and the *icmL*::Tn mutant.
(TIF)

Figure S2 **Relationship between infection of BMMs and translocation.** BMMs from C57BL/6 mice were seeded into 96 well trays and infected with *C. burnetii* NM pBlaM-77 at several multiplicities of infection (MOIs) at 24 h post-infection triplicate wells were analysed for translocation of BlaM-77 using the CCF4-AM substrate. Translocation was quantified visually by examining at least 300 cells per well and recording the percentage of BMMs that were translocation positive (white bars). Replicate wells were fixed with 4% PFA and the bacteria were stained with mouse anti-*C. burnetii* (1∶5000) and Alexa Fluor 596 anti-mouse before secondary fixation and permeablization. Samples were then stained with rabbit anti-*C. burnetii* (1∶10000) and Alexa Fluor 488 anti-rabbit. This enabled the intracellular bacteria to be identified as they fluoresce green only. Approximately 300 cells per well were examined for intracellular *C. burnetii* and the proportion of infected BMMs was calculated (black bars).
(TIF)
