# Supplementary figures and images for: Effector Protein Translocation by the Coxiella burnetii Dot/Icm Type IV Secretion System Requires Endocytic Maturation of the Pathogen-Occupied Vacuole
Source: PLoS One. 2013 Jan 17;8(1):e54566. doi: 10.1371/journal.pone.0054566 (PMC3547880; doi:10.1371/journal.pone.0054566)

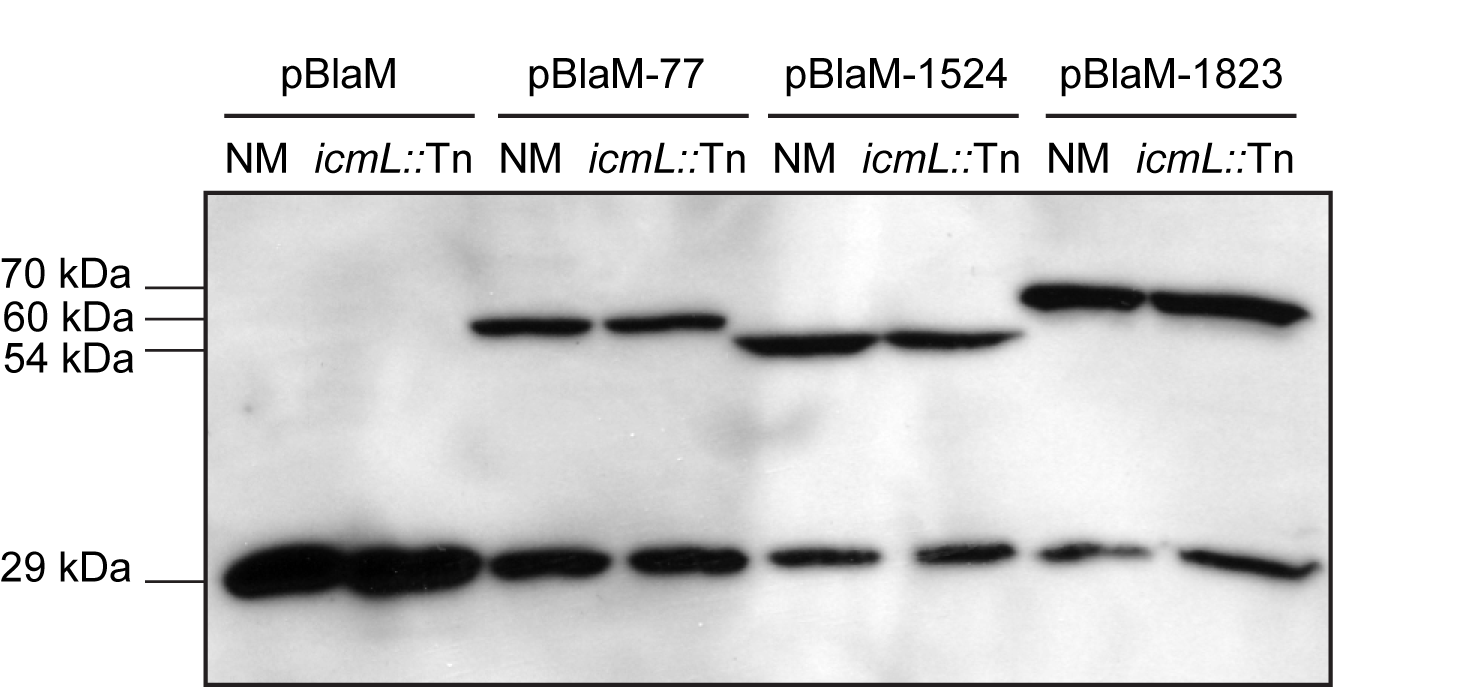

Supplement: Figure S1 — BlaM-effector fusion proteins expressed in C. burnetii . Immunoblot analysis of C. burnetii NM phase II and the icmL::Tn mutant following the introduction of pJB-CAT-BlaM (BlaM) and BlaM-effector fusion constructs (pBlaM-77, pBlaM-1524 and pBlaM-1823). Lysate from stationary phase ACCM-2 cultures were probed with anti-BlaM (1∶5000) and the highlighted bands demonstrated expression of BlaM (29 kDa), BlaM-77 (60 kDa), BlaM-1524 (54 kDa) and BlaM-1823 (70 kDa). Importantly, expression of each reporter protein was comparable in C. burnetii NM phase II and the icmL::Tn mutant. (TIF) [file pone.0054566.s001.tif]

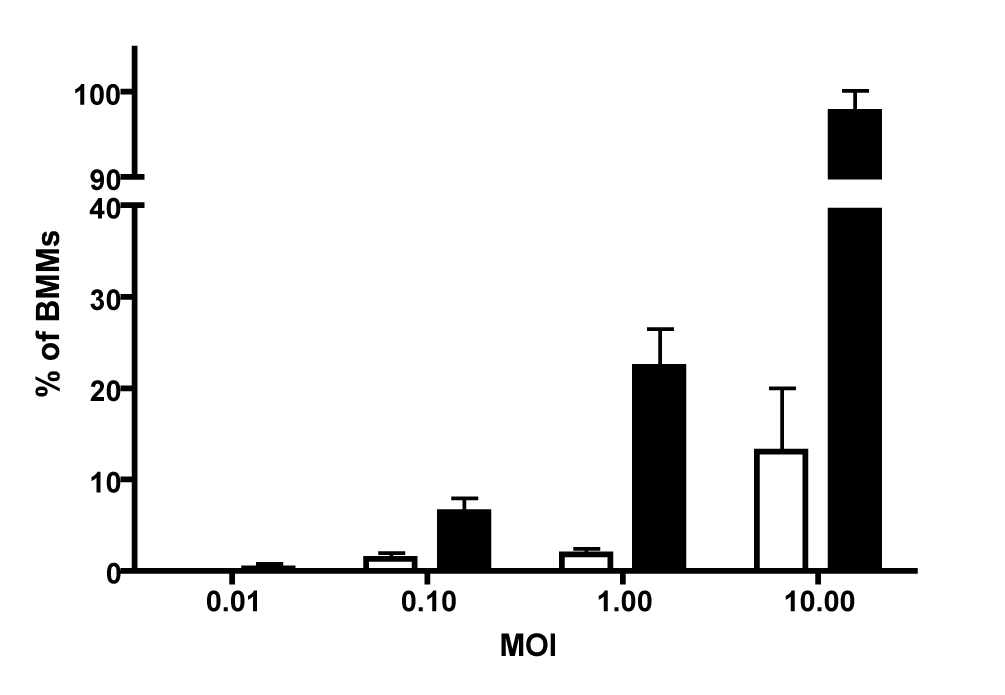

Supplement: Figure S2 — Relationship between infection of BMMs and translocation. BMMs from C57BL/6 mice were seeded into 96 well trays and infected with C. burnetii NM pBlaM-77 at several multiplicities of infection (MOIs) at 24 h post-infection triplicate wells were analysed for translocation of BlaM-77 using the CCF4-AM substrate. Translocation was quantified visually by examining at least 300 cells per well and recording the percentage of BMMs that were translocation positive (white bars). Replicate wells were fixed with 4% PFA and the bacteria were stained with mouse anti-C. burnetii (1∶5000) and Alexa Fluor 596 anti-mouse before secondary fixation and permeablization. Samples were then stained with rabbit anti-C. burnetii (1∶10000) and Alexa Fluor 488 anti-rabbit. This enabled the intracellular bacteria to be identified as they fluoresce green only. Approximately 300 cells per well were examined for intracellular C. burnetii and the proportion of infected BMMs was calculated (black bars). (TIF) [file pone.0054566.s002.tif]
